# “I Just Stopped Taking PrEP Without a Reason”: Understanding PrEP Decision-Making Amongst South African Adolescent Girls and Young Women (AGYW)

**DOI:** 10.1007/s10461-025-04994-3

**Published:** 2025-12-21

**Authors:** Zoe Duby, Kate Bergh, Kim Jonas, Mari Lotvonen, Caroline Kuo, Catherine Mathews

**Affiliations:** 1 Health Systems Research Unit, South African Medical Research Council, Cape Town, South Africa; 2 School of Public Health, University of Cape Town, Cape Town, South Africa; 3 Networking HIV/AIDS Community of Southern Africa (NACOSA), Cape Town, South Africa; 4 American University, Washington, USA; 5 Department of Psychiatry and Mental Health, University of Cape Town, Cape Town, South Africa

**Keywords:** PrEP, Discontinuation, HIV prevention, Adolescent girls and young women, South Africa, Decision-making

## Abstract

Pre-exposure prophylaxis (PrEP) use is not a once-off bio-behavioural strategy but part of ongoing HIV prevention decision-making. We explored PrEP decision-making through qualitative interviews with 68 adolescent girls and young women (AGYW) aged 15–24 years from communities in which a large-scale combination prevention intervention supporting daily oral PrEP uptake for AGYW in South Africa was implemented. Thematic analysis of transcripts was informed by a social-ecological framework. AGYW’s decisions to initiate, continue, discontinue, or refuse PrEP were shaped by intersecting individual, interpersonal, health system, and contextual factors. At the individual level, self-motivation for HIV protection and distrust of partners supported initiation and continuation, reinforced by regular HIV-negative results, while side effects, dislike of daily pills, and perceived contraindications drove discontinuation. Decisions to refuse PrEP centred around low perceived HIV risk, fear of side effects, and aversion to daily tablets, although many expressed conditional interest in future long-acting formulations. Interpersonally, disclosure to and encouragement from family, partners, or friends enabled continuation, whereas inability to disclose, limited social support, and relationship changes undermined adherence. At the health system level, provider engagement, support, and perceived confidentiality of services and packaging were pivotal. Contextually, PrEP-related stigma constrained use, while living in high HIV-prevalence, unsafe communities heightened risk perception and encouraged uptake. PrEP use among AGYW is dynamic and temporal, shaped by relationships, communication and disclosure, embodied experiences, stigma, and service experiences. There is a need for tailored, responsive prevention counselling and service delivery to support AGYW’s PrEP initiation, persistence, and informed decisions about sexual health.

**Supplementary Information:**

The online version contains supplementary material available at https://doi.org/10.1007/s10461-025-04994-3.

## Introduction

 It has been almost a decade since adolescent girls and young women (AGYW) were identified as a population that should be prioritised for pre-exposure prophylaxis (PrEP) delivery by both the World Health Organisation, and the South African government’s National Department of Health (NDoH) in the National Strategic Plan for curbing HIV, TB and STIs: 2017–2022. Subsequently the national guidelines for daily oral PrEP for HIV prevention were updated to include AGYW [1]. Whilst oral PrEP has been shown to be highly efficacious, its effectiveness is dependent on effective use, specifically uninterrupted use prior to time of exposure [2]. A user must take a daily pill on at least four days in a week to be protected [3]. Increasing evidence shows that among South African AGYW, levels of PrEP uptake are high, but adherence and persistent use remain a challenge, with a significant proportion of AGYW who initiate PrEP discontinuing within the first three months [1, 2, 4–7]. This paper describes the experiences of daily oral PrEP only, because the options for PrEP were limited to this modality during the study period and in the study setting. PrEP options have now expanded globally to include injectable long-acting cabotegravir (CAB-LA) – consisting of cabotegravir and administered every two months by a clinician – and the monthly Dapivirine vaginal ring (DVR). In South Africa, these modalities are being piloted but are not yet widely available [8, 9].

Decision-making processes about HIV prevention are a critical aspect of enacting HIV preventative behaviours. The decision to use an HIV prevention method is individual and affected by cognitive-psychological factors such as perceived relevance, risk perception and self-efficacy, but additional interpersonal, contextual, and structural processes influence decision-making and the agency an individual has to enact associated behaviours [10, 11]. The development and efficacy of novel prevention methods, and ensuring access to them is undeniably important, but the product user’s decision-making processes determine whether or not they engage with the technology [10]. In recent literature there has been a shift towards framing PrEP use as a multi-phase continuum comprising deciding to protect oneself from HIV infection, choosing and accessing a method, initiating use, using, and discontinuing use. At each of these phases on the continuum, decisions are made based on a variety of factors inclusive of somatic and lived experiences, perceptions of efficacy, context, interpersonal relationships and perceived views of others [12–14]. The concept of patient ‘journeys’ has been employed to better understand barriers, facilitators, experiences, interactions with services and outcomes for individuals as they enter, navigate, experience and exit services in a health system, in order to inform the design and provision of responsive and context-specific strategies for improving patient healthcare experiences and outcomes [15].

Like contraceptive use, PrEP use is not a once-off preventive intervention, but rather an on-going iterative ‘journey’ of decision-making, embodied experience, and health service interaction that plays out over the course of an individual’s sexually active life course [12, 14, 16]. In the context of PrEP, a prevention medication rather than a treatment, the user is not unwell, but still has to enter into and engage with the health system. However, unlike a patient in chronic care, the need for PrEP differs not only between individuals, but for the same individual over time, as they go through ‘seasons of risk’ [17]. By examining a PrEP user’s experiences and decision-making processes, we can help to identify bottlenecks and gaps in PrEP service provision, evaluate the continuity of PrEP services, understand how PrEP users navigate PrEP services, in order to tailor responsive service delivery [15].

Whilst increasing uptake and reducing barriers to access of PrEP for AGYW is critical, it is also necessary to consider the continuum of uptake, use, and discontinuation, and on-going decision-making around HIV prevention, as well as the multi-level factors that influence decision-making over the sexually active life course. There is currently relatively scant data available on AGYW decision-making around when to use PrEP, and whether to continue or discontinue using it, and a lack of evidence-informed strategies that successfully support AGYW in persisting with PrEP use, and their on-going engagement with PrEP services [2]. A deeper understanding of the individual, interpersonal, socio-cultural, and contextual factors that impact AGYW’s on-going PrEP decision-making and effective use is needed [6].

**Conceptual Framework**: Adaptations of the social-ecological model, Bronfenbrenner’s Ecological Systems Theory of Human Development (1979) are used to examine interactions and relationships between individuals and their social and environmental context. These models can be applied to health-related behaviours due to the way in which they account for how decisions like PrEP uptake are shaped by interacting factors at the (1) individual level such as attitudes, beliefs and knowledge; (2) interpersonal relationships including those with peers, partners and families; (3) health system aspects including PrEP service provision; and (4) contextual and community contexts.

Qualitative research can help to understand AGYW’s perceptions of their needs and decision-making processes throughout their PrEP journeys, inclusive of their initial PrEP awareness, uptake and initiation, through adherence/persistence, and possible discontinuation [18]. This study provides a valuable illustration of the lived realities and subjective experiences of AGYW as they navigate their PrEP decision-making journeys, thus deepening our understanding of why South African AGYW start, stop, or continue using PrEP. While most of the data relates to daily oral PrEP, findings also provide insight into the implementation of other forms of PrEP including vaginal rings and long-lasting injectables that are likely to become increasingly available in the South African setting.

## Methods

Qualitative in-depth interviews were conducted with AGYW from communities in which a large-scale combination HIV intervention, the My Journey Programme, was implemented. Participants were recruited from a larger evaluation study of the My Journey Programme in which a household survey was conducted (https://www.samrc.ac.za/intramural-research-units/HealthSystems-HERStory). Survey participants were invited to participate in additional telephonic in-depth interviews. Those willing provided their cell phone numbers and later were contacted by an interviewer.

The My Journey Programme, a combination HIV prevention intervention for AGYW in South Africa, started in 2016 and is on-going. The My Journey Programme is funded by the Global Fund to Fight AIDS, TB and Malaria, and implemented by three Principal Recipient organisations that in turn contracted sub-recipients to implement the intervention components. One of the aims of the combination intervention is to reduce HIV incidence. AGYW are introduced to the programme through a number of entry points and referred for services via two main components called the Core and Layered Services which are delivered by funded sub-recipients in schools, Technical Vocational Education and Training (TVET) colleges, dedicated safe spaces in communities, and mobile clinics that deliver clinical HIV and SRH related services. Layered services are categorised into biomedical, behavioural and structural services. In addition, some layered services are delivered by unfunded external service providers such as government health, education or social development providers, in their own settings via referrals from the funded sub-recipients. The approach of the My Journey programme is to leverage these existing services rather than set up parallel and less sustainable services.

The My Journey Programme aimed to provide PrEP services in alignment with the NDoH’s scale up and roll out plans for AGYW, through supporting the work done in state sector health facilities by offering community-based services. Mobile clinic and outreach services were equipped to initiate daily oral PrEP for AGYW who test HIV negative at these sites. The My Journey programme comprised PrEP demand creation activities, including distribution of information, education and communication (IEC) materials designed by the NDoH, as well as distribution of materials through community venues, mobile services and other venues as possible. The programme also aimed to increase PrEP demand through social media communication, meeting with community advisory boards to introduce PrEP, training on PrEP for educators within the My Journey programme and those within the Adolescent and Youth Friendly Services (AYFS) with guidance from NDoH, discussion on PrEP within men’s dialogues with the aim of educating males as partners of females, holding PrEP information days for community members, and doing PrEP advertising during events. As part of the programme implementation, implementers co-developed provincial PrEP implementation plans with NDoH, to ensure harmonisation of the Global Fund PrEP targets, roll out and delivery methodologies with Departmental procurement, strategies and protocols.

**Fig. 1:**
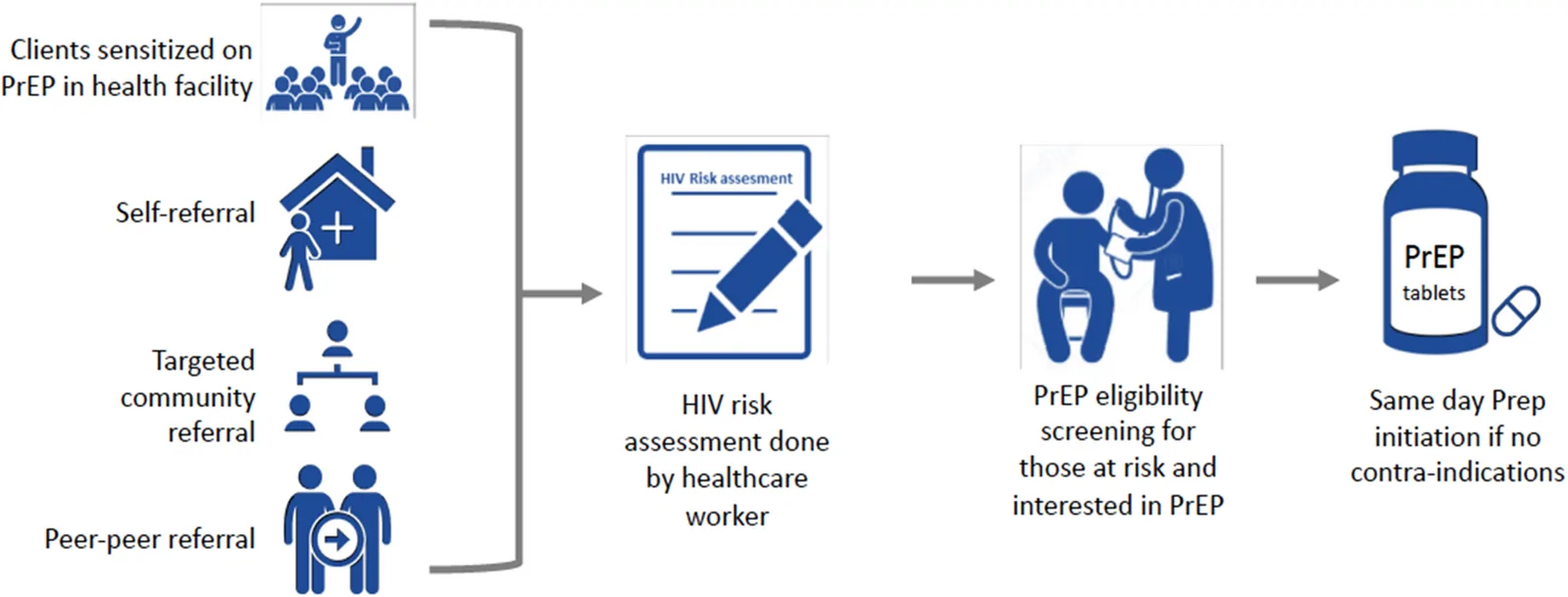
My journey programme PrEP delivery flow chart

As illustrated in Fig. 1, the My Journey Programme outlined PrEP delivery to AGYW starting through several referral points, which then led AGYW to undergo an HIV risk assessment inclusive of an HIV rapid test confirming HIV negative status, followed by a PrEP eligibility screening. Those AGYW who tested HIV negative, were assessed to be at risk and interested in PrEP were then offered same day PrEP initiation to minimize the loss of uptake.

### Data Collection

As shown in Table 1, qualitative interviews were conducted telephonically with a total of 68 AGYW aged 15–24 years (thirteen adolescent girls aged 15–17 years, and 55 young women aged 18–24 years). Participants were sampled in subdistricts in which the My Journey Programme was implemented (see Table 1).

**Table 1:** Study sample

Province	Sub-district	District	15–17 years	18–24 years	Total
KwaZulu-Natal	AbaQulusi	Zululand	0	3	3
	uMhlathuze	King Cetshwayo	0	4	4
Mpumalanga	Mbombela	Ehlanzeni	0	11	11
	Govan Mbeki	Gert Sibande	0	3	3
Eastern Cape	Nelson Mandela C	Nelson Mandela Bay Metro	0	1	1
	Nyandeni	Oliver Tambo	1	1	2
Free State	Dihlabeng	Thabo Mofutsanyana	2	2	4
	Setsoso	Thabo Mofutsanyana	3	4	7
Gauteng	Tshwane 1	Tshwane	2	6	8
North West	Rustenburg	Bojanala	2	8	10
Western Cape	Klipfontein	City of Cape Town	3	12	15
**Totals**			13	55	68

Interviews took place in participants’ choice of language (isiZulu, Sesotho, Setswana, Afrikaans, isiXhosa or English), at a time convenient for the participant, following a semi-structured interview guide, and lasted between 20 and 120 min. Female researchers fluent in the study languages, with tertiary qualifications ranging from Bachelor’s to Masters’ degrees and trained on qualitative interview techniques and good research ethics conducted the interviews. The researchers did not have any prior or existing relationships with study participants. Prior to conducting the interviews, interviewers checked with participants if they were in a safe, private and comfortable place to continue with the interview. Interviews were audio recorded with participants’ consent, labelled with unique codes, and saved in a secure, password protected online drive.

All AGYW participants were asked questions related to PrEP (see Supplementary information for Interview Guide questions relating to PrEP), inclusive of questions on PrEP knowledge and experiences interacting with PrEP services. Those participants who had ever taken PrEP were asked about their experiences with PrEP initiation, use, adherence and/or discontinuation. Those participants who had been offered PrEP but refused it, were asked about their decision-making processes.

### Data Analysis

Audio recordings of interviews were directly translated from their original language into English transcripts and were anonymised to protect participant confidentiality. A Collaborative Qualitative Analysis process grounded in thematic analysis was employed, in which English transcripts were analysed collaboratively by three analysts using shared password-protected online summary memos and coding documents. These were continuously updated to document transcript coding, emerging themes, and analysts’ reflections on the data [19, 20]. The analysis process evolved iteratively through a combination of deductive and inductive approaches reflecting the study’s key objectives and topics that emerged through reading the data as well as through dialogue and consensus [21]. A set of pre-determined deductive code types based on the topics included in the interview guides were built upon through the inductive development and refinement of codes. The initial analysis structure and set of deductive codes was derived from the interview topic guides, which had been developed based on the key research objectives. These deductive codes captured pre-defined areas of inquiry corresponding to the core domains of the guides. As transcripts were reviewed, these codes were iteratively refined and expanded through inductive coding to capture unanticipated themes and insights emerging from participants’ narratives. In addition to descriptive themes, pattern themes, which achieve a greater level of abstraction, were used to start linking themes and topics together in order to explore relationships in the data. As analysis progressed respondents were sorted into three groups: currently using, discontinued, and refused. Analysts developed analysis memos for each respondent based on their category.

## Findings

### Description of Participants

In our analysis, we categorised participants into three categories based on their self-reports and narratives: (1) Currently using PrEP; (2) Discontinued PrEP (those who had initiated PrEP via programmes being evaluated and had discontinued by the time of the interview); (3) Refused PrEP. Of the total 68 AGYW we interviewed, 5 were currently using PrEP, 8 had initiated on PrEP but had discontinued and the remaining 55 had refused PrEP. Data presented below comprise quotations from interviews in which pseudonyms are used for AGYW who are directly quoted, as detailed in Table 2. Figure 2 illustrates the multi-level factors that influenced AGYW PrEP decision-making and use arranged by social-ecological levels.

**Table 2:** Quoted participants’ pseudonyms

Pseudonym	Group	Province	Sample group
Andiswa	Currently using	Mpumalanga	18–24 years
Zandi	Currently using	Mpumalanga	18–24 years
Buhle	Currently using	Mpumalanga	18–24 years
Koli	Currently using	North West	18–24 years
Vundli	Currently using	Eastern Cape	18–24 years
Lebo	Discontinuer	KwaZulu Natal	18–24 years
Jabu	Discontinuer	Gauteng	18–24 years
Sethu	Discontinuer	Mpumalanga	18–24 years
Yolisa	Discontinuer	Mpumalanga	18–24 years
Ntombi	Discontinuer	North West	18–24 years
Bontle	Discontinuer	North West	18–24 years
Dineo	Discontinuer	North West	18–24 years
Sindi	Discontinuer	KwaZulu Natal	18–24 years
Karabo	Refuser	North West	15–17 years
Michelle	Refuser	Western Cape	15–17 years
Fundiswa	Refuser	Mpumalanga	18–24 years
Annie	Refuser	Gauteng	18–24 years
Sesi	Refuser	Free State	18–24 years
Khanya	Refuser	KwaZulu Natal	18–24 years
Lerato	Refuser	KwaZulu Natal	18–24 years
Aliya	Refuser	Western Cape	18–24 years
Esther	Refuser	North West	18–24 years
Zainab	Refuser	Western Cape	18–24 years

In terms of initial encounters with PrEP amongst currently using and discontinuers, and where they accessed PrEP, the two main sources were: (1) at a government clinic when accessing contraceptives, when attending antenatal care services, or when testing for HIV with a partner who tests positive; (2) PrEP programmes in the community, or at school.

### ***Findings by*** social-ecological ***Levels***

**Fig. 2:**
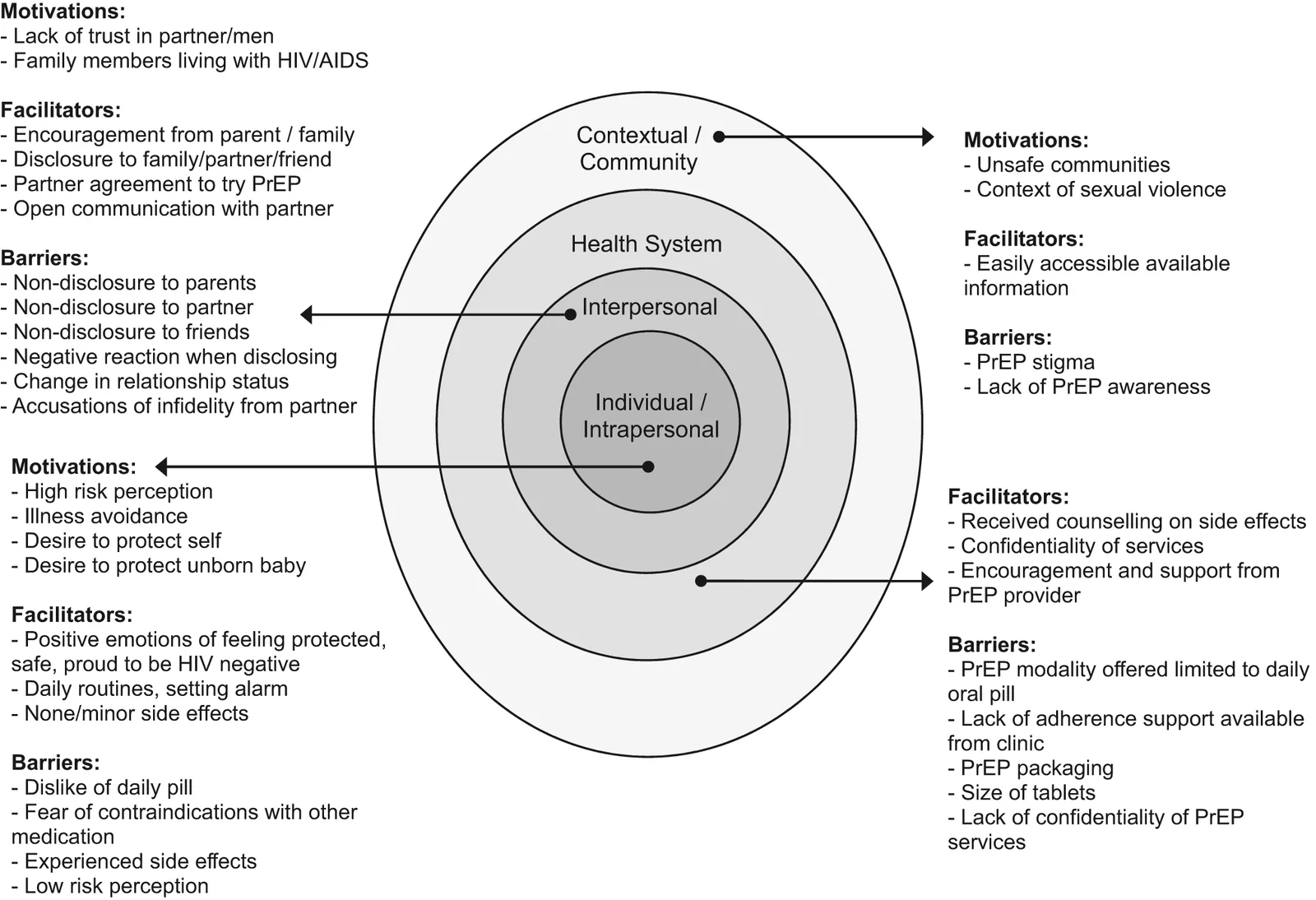
Motivations, facilitators and barriers to PrEP use presented by socio-ecological levels

## Individual/Intrapersonal

### Risk Perception

Self-motivation to protect oneself was a key factor determining the decision to initiate PrEP use, as explained by Zandi: “I felt great (when I first heard about PrEP) because I knew that I will be protected, even if my partner does silly things behind my back”. Motivations to initiate PrEP amongst discontinuers were similar to those currently using PrEP, with self-protection being key. Lebo initiated PrEP while she was pregnant: “because I was protecting my baby”.

For those AGYW currently using PrEP, the feeling of being protected and safe was also a major motivation to continue taking PrEP. Continued testing negative for HIV made AGYW feel happy and proud, and motivated to persist with PrEP use, as narrated by Vundli: “I was happy (to receive PrEP) because I knew it is going to help me with life and my protection… I feel protected and happy because I know I will stay negative… Ever since I started taking PrEP I am not infected with HIV and am proud to test (HIV negative) every time”. Taking responsibility for one’s own health was described as critical by Koli: “as a person you must do (what you can) so that you cannot get diseases”.

Amongst those AGYW who had heard about PrEP, and been offered it, but had decided not to take it, having a low-risk perception was one salient reason provided. Low-risk perception was particularly evident among adolescent girls aged 15–17 years, such as Karabo: “I don’t (want to use PrEP)… because I know I won’t get any infections… because I’m always at home”. Of those AGYW who had decided not to take PrEP, several articulated their intentions to use PrEP in the future, stating that they did not feel they needed it right now, or that they were not quite ready to initiate, as Khanya indicated: “My thoughts were that I was going to take it someday, but I did not take it instantly… I will (use PrEP in future)… to protect myself”. PrEP was regarded as a useful product, given the importance of looking after one’s own health and difficulties trusting sexual partners, as articulated by Lerato: “I see it as a good product… it prevents you from getting infected with HIV even if you have partner that is infected with HIV, still it protects you”.

### Side Effects

Side effects were a key factor determining the decision to continue or discontinue daily oral PrEP use. Some of those who were using PrEP at the time of the interviews explained that whilst they may have had some initial side effects, these were considered minor, or had subsided, as described by Andiswa: “at first, I got very dizzy taking the pills, and when I asked about it, I was told it is just the side effects… I feel alight and normal now”. Side effects were a key reason for discontinuation. Feeling ill from taking daily oral PrEP made it hard to be motivated to keep taking it daily, as Jabu explained: “you have the feeling that you are sick, why I am taking this thing every day?”. For some, outwardly visible side effects were also a reason to be encouraged to discontinue PrEP use by parents and partners as Yolisa experienced: “(my family) said I should stop using it if it is not treating me good… my partner said I should stop using it if they are not good for me”. Ntombi recognised that not everyone responds the same way to oral PrEP and that for some, the side effects are not as serious: “not all things settle the same way in people’s body, my only problem is the headaches”.

Additionally, fear of side effects was the most commonly cited reason for choosing not to use PrEP amongst the refuser group. This quotation from Michelle illustrates the fears relating to fears of potential side effects outweighing risk perception: “To be honest… I would like to go on it (PrEP) because I am sexually active but… (I decided not to use it because I was) afraid of a lot of stuff… a lot of girls had different reviews about it… some, their hair fell out, and some of them lost weight… everyone gave different reviews, and it made me feel anxious. I was scared about how it might affect my body”. It was suggested that in order to initiate PrEP in the future, one would need comprehensive information about how it works, as stated by an Aliya: “If I know what it does to your body, then I’ll consider it”. Among those AGYW who were offered PrEP but refused to take it, some admitted they had been intrigued by it, and considered taking it, but that the decision not to take it outweighed their curiosity, as explained by Esther: “I do have conflicting (feelings), I’ve never tried PrEP, I wanted to but I never tried it… I have confused feelings towards it even now… I don’t know [chuckles]. I’m just… it has side effects they say, so I’m avoiding those things… Like nausea and dizziness and vomiting… That’s why I’m not even considering taking it”.

### Dislike of Oral Daily Pill

Other barriers to daily oral PrEP persistence at the individual level amongst those who discontinued PrEP included dislike of the daily pill burden, the fact that having to take daily medication made you feel like you were an unwell person, as voiced by Dineo: “I stopped using them (PrEP)… I felt like I was sick… because of the pill you take every day, every day”. Concerns about contraindications with other medications was another reason to discontinue, as recounted by Bontle “(I stopped taking PrEP because) I was taking something else (another medication) and I heard you’re not supposed to mix other things with pills… so I thought let me stop using PrEP”.

Reasons for refusal to take PrEP specific to the oral daily tablet as a delivery mechanism included perceived medication burden. The idea of having to take a pill daily was a major off-putting factor for oral PrEP. Another reason cited for choosing not to take PrEP was the dislike of oral tablets, as articulated by Fundiswa: “I won’t use PrEP in the future… My problem is taking pills every day”. For some AGYW in the refusers group, it was suggested that if PrEP was available in the form of a longer-acting method such as an injection, then they’d consider it, such as Annie: “I was offered PrEP (but didn’t take it)… I’m not a consistent person. I know I would forget the pills… I don’t think I’d be able to take pills every day. Basically, if it was a one-time shot, or one-time pill… I’d take it”.

### Interpersonal

#### Disclosure and Encouragement

For those participants currently using PrEP at the time of being interviewed, the successful disclosure of PrEP use to family, partners and friends was a critical enabler, as articulated by Andiswa: “they did not have a problem with it… they told me it was a good idea”. Zandi also illustrated how disclosure enabled support and encouragement from family and partners: “I talked to my mother… she asked me about this PrEP and I explained to her… She encouraged me to continue if I feel it will help me… It was easy because we chat about anything and everything”. Conversely, not being able to disclose to family and partners negatively affected the ability to continue taking PrEP.

### Relationship Status

Lack of trust in a sexual partner was a motivation to use PrEP as Zandi explained: “people are not trustworthy, I will not know what is happening in my absence”. Changes in relationship status also affected the motivation to continue taking PrEP, either with those that were no longer in relationships feeling PrEP was unnecessary, as described by Ntombi: “I’m no longer dating the person I use to date… I think I’ll give myself a break from dating… if I’m not dating I do not see the need (to take PrEP)”. Ending a relationship with a partner known to be living with HIV was also a reason to discontinue.

### Health System Level

At the health service level, feeling encouraged and supported by the PrEP provider was an enabler, as voiced by Vundli: “The person that was giving me PrEP was nice, also encouraging me not to stop using PrEP”. Other enablers described including perceiving PrEP services as confidential, and easy access to information on PrEP.

In terms of health service barriers, the feeling of not being supported or monitored by health service providers was a motivation to discontinue, as described by Lebo: “At the clinic they did not care whether you take it or stop, they don’t check if I’m still taking it or not… I even asked for it myself… I used to constantly remind them (healthcare workers) about giving me the pills”. Andiswa also experienced health system related barriers to continuing with her PrEP use, explaining that she was unimpressed with the confidentiality of the PrEP services she had received at the clinic: “I felt like people are looking at me… at first, the PrEP was issued to me in public in front of everyone… a crowd of people could see you. Before we were mixed with those (patients) collecting HIV treatment”. However, Andiswa explained that the confidentiality of services improved which made her more comfortable receiving PrEP and able to continue using it: “now it is issued in a private room and no one can see”.

The packaging of daily oral PrEP was one aspect of PrEP stigma that was a barrier to continuation, as explained by Andiswa: “They (PrEP containers/packets) are almost the same (as ARVs)”. For those AGYW who did initiate PrEP, but then discontinued, PrEP stigma related to daily oral PrEP tablet packaging, leading to PrEP being confused with ARVs was also cited as a barrier. Also related to oral PrEP was concern around the size of the tablets themselves, as Sesi describes: “They offered it (PrEP) to me… I did not take it because I saw that they are these big pills”.

### Contextual/Community Level

#### Community Safety

Community safety contexts played a role in AGYW PrEP decision making. High HIV risk-perception based on an awareness that the communities where AGYW live have high HIV prevalence and are unsafe, was a key motivation to use PrEP, as voiced by Buhle: “What made me decide to use PrEP… I do not want to get sick due to HIV because I had seen my relatives who are living with HIV, so I did not want to see myself living that kind of life… it was my own decision… lots of things are going on around us. We are young so we go out for drinks and anything can happen on the streets so I feel safe when I am taking PrEP… I like it”.

### PrEP Stigma

In addition to PrEP stigma related to the packaging, Ntombi explained another aspect of PrEP stigma due to the associations of PrEP with promiscuity: “when you’re taking PrEP most people think that you are promiscuous like maybe you are not dating just your boyfriend”. Fear of anticipated stigma and judgement was also the reason Dineo chose not to disclose to her parents: “I was scared that they would judge me”. Another fear was being accused by partners that taking PrEP was a sign of infidelity, which was Dineo’s reason not to disclose to her partner: “I was scared that he would ask me questions whether I’m sleeping with other people”. A lack of knowledge and information about PrEP at the community level was also cited as a barrier to use, heightening AGYW fears of stigma and judgement, as explained by Ntombi: “most people know about ARVs and not PrEP so the embarrassing part is just there”.

## Discussion

The findings from this study highlight the complexity of AGYW decision-making processes relating to PrEP, demonstrating that it is not only the decision made at a singular point of time, regarding whether or not to use PrEP that matters, but an on-going decision-making process influenced by multi-level factors, as well as subsequent experiences of using the PrEP modality. In the narratives articulated in our study, AGYW’s PrEP use decisions reflected a balance between temporal perceived personal HIV risk and protection benefits versus concerns about side effects, pill-taking burden, social support and stigma, with expressed demand for discreet, long-acting alternatives. As the decision to use PrEP is nonlinear and context-dependent, AGYW need on-going support in their decisions around starting, stopping, and continuing to use PrEP, as their life and relationship situations shift and change. These findings can help to inform the development of tools to support AGYW PrEP decision-making by shedding light on the PrEP experiences and decision-making processes of AGYW who have interacted with PrEP services across South Africa.

Self-motivation related to HIV risk perception was a key component for AGYW respondents in our study to start using and continue using PrEP. Those AGYW interviewed who managed to continue with their PrEP use were motivated by remaining safe, protected and healthy, particularly when in relationships characterised by a lack of trust, and when living in high-risk settings. Amongst those respondents who were currently using PrEP, motivations to persist with PrEP use were tied to a strong sense of feeling protected and safe in contexts of high HIV prevalence and sexual violence, leading to a strong motivation to continue using it. Another recent study in South Africa found that even AGYW who were not sexually active were motivated to use PrEP due to fear of sexual assault in communities with high levels of sexual violence [22]. Self-motivation is linked with risk perception, feelings of being vulnerable to HIV infection, and a sense of hope for the future [4, 18].

However, as evident from those AGYW in our study who discontinued PrEP use, motivation and perceived risk are insufficient to ensure persistence. Findings from our study show that self-protection was a key motivation to initiate PrEP use both among those AGYW currently using PrEP and those who had discontinued. This demonstrates that motivation alone was not sufficient for persistence, and that the capacity of AGYW to sustain PrEP use is shaped by individual emotional and cognitive factors, including fear, hope and risk-perception, interacting with interpersonal factors such as social support and shifting relationship dynamics. The disconnect between intention and action has previously been described in a study examining PrEP use amongst AGYW in Kenya [23]. It has been suggested that adolescents and young people engaging in decision-making around HIV prevention may experience negative emotions such as fear, discomfort and anxiety, which impede rational decision-making and successful uptake and effective use of prevention products and services [11]. Given the critical role that hope for the future plays in adolescents’ enactment of health promoting behaviours such as HIV prevention, interventions should aim to incorporate efforts to foster hope amongst AGYW living in these communities in South Africa [24].

Linked to individuals’ risk perceptions and motivations, interpersonal factors such as changes in relationship status were listed as a factor influencing PrEP discontinuation, with no longer being in relationship being cited as a reason to discontinue PrEP. Individual decision-making and enactment of HIV preventative behaviours are strongly influenced by emotional processes related to perceptions of risk, which may fluctuate in salience and priority for the individual dependent on interpersonal and relationship contexts, and long/short terms goals [11]. The deliberation of risks and benefits that individuals engage in when evaluating HIV prevention options does not always follow rational decision-making and is affected by emotional state and context, highlighting the need to ensure on-going access to integrated SRH services that are responsive to shifts in relationship status and seasons of risk, which may help to improve uptake, acceptability and efficacious use [11, 24, 25]. Considering the challenges in PrEP continuation and persistence by AGYW PrEP users, aiming to achieve prevention-effective adherence, which refers to periodic use of PrEP during seasons of risk (periods of potential HIV exposure), may be a helpful strategy but would require the provision of counselling and support on how to start and stop PrEP safely, to be successful [26].

Support and disclosure were also key influencing factors at the interpersonal level. Many of those respondents currently using PrEP reported that they received support from family members and/or partners for their PrEP use. Among AGYW who successfully continued using PrEP in our study, the successful disclosure of PrEP use to family members (particularly mothers), partners and friends was a critical enabler to persistence, enabling support and encouragement for PrEP use. However disclosure itself was not always an enabler to continuation if the reaction received upon disclosure was a barrier to PrEP use. Amongst discontinuers in our study, some had disclosed their oral PrEP use to partners who were not supportive, and some had disclosed to family who supported their decisions to discontinue after experiencing side effects. Expanding on findings from a study conducted in South Africa’s Eastern Cape province, our findings highlight that one key difference between those that continued using PrEP and those who discontinued, was social support and encouragement for PrEP use [3]. Evidence from both South Africa and other sub-Saharan African settings has outlined lack of disclosure about PrEP use as a key barrier to persistence of use; successful disclosure to family substantially increases the likelihood of successful PrEP adherence [5, 27]. Given that adolescents are likely to live with family members, parents and caregivers have a strong influence on their behaviour and decision-making, and are an important source of social support, and have been shown to be a critical enabler in adolescents’ effective PrEP adherence and persistence [28, 29]. Our findings add to the evidence base highlighting the critical need for social support interventions to bolster the support that AGYW receive from their families and household members for their PrEP use, recognising the role that mothers in particular play in facilitating PrEP decision-making and continuation amongst AGYW [5, 27].

Some of the our study respondents currently using PrEP who had disclosed their PrEP use reported that they valued PrEP and its protective effects so highly that they shared their PrEP supplies with friends or family members they deemed to be at risk in the desire to protect them too. PrEP sharing behaviours have been described in previous studies [5, 30], suggesting that further research is required to understand PrEP sharing, its implications, and how it could be capitalised upon to reduce PrEP stigma, increase PrEP acceptability and uptake, create an enabling environment for PrEP use, and thus enhance AGYW PrEP persistence.

A key factor intersecting at the individual somatic experience level and the health system level in terms of drug formulation related to side effects from PrEP use. Side effects were a central determinant of adherence across participants, underscoring the embodied nature of PrEP experiences. Unpleasant side effects described by participants included nausea, pain, headaches, vomiting, fatigue, dizziness, and appetite increase. Those AGYW currently using PrEP and those who discontinued shared many of the same initial use experiences, with most describing side effects. One difference between these groups was that amongst those currently using PrEP, side effects were either not considered serious or subsided quickly, whereas for those who discontinued, the side effects were considered too unpleasant to endure. Among those AGYW in our study who had started taking PrEP but then discontinued use, the most salient barrier to continuation was the experience of side effects. As has been discussed in the context of contraceptive side effects, whether or not the side effects described by AGYW PrEP users are clinically ‘provable’ or objective, adherence is impacted by the lived reality and embodied experiences of using PrEP [14]. For this reason, PrEP services need to include specific youth-tailored counselling around potential and experienced side effects, in a manner that is respectful of and responsive to the user’s experience [14, 31]. Studies have suggested that oral PrEP is clinically ‘safe’ for adolescents and young people to use, and ‘relatively well tolerated’, with temporary ‘self-limiting’ side effects diminishing after the first few weeks of use [32, 33].

Although from a clinical perspective the side effects that AGYW respondents in our study described, such as headaches, changes in appetite, and drowsiness, may be considered minor, from an adolescent user’s perspective these somatic experiences are likely to be uncomfortable and concerning, leading to discontinuation. Evidence suggests that using peer-led PrEP adherence clubs and peer support networks in which current PrEP users share side effect experiences and coping strategies can alleviate concerns and improve persistence [31, 34]. It has also been suggested that amongst socio-economically restricted communities such as those in our study, where food insecurity is prevalent, side effects may be exacerbated by hunger [33]. This would be an important consideration in PrEP delivery across South Africa and the broader sub-Saharan African region.

The physical packaging of oral PrEP was a factor affecting PrEP use intersecting individual, interpersonal, health system and community levels. As found in our study and in prior research, the packaging of oral PrEP (pill bottles, tablet packets) is also a concern among AGYW who fear PrEP stigma and association with ARVs, indicating that branding and packaging of oral PrEP for AGYW in less conspicuous ways – for example in innocuous cosmetic/makeup bags or similar to simple medications – may help to allay AGYW fears relating to PrEP stigma [35, 36]. Offering AGYW the opportunity to cycle on and off PrEP during seasons of risk may also help to differentiate PrEP from ARVs in users’ and community understanding [26]. It is worth noting that the central role that disclosure plays in successful oral PrEP persistence among AGYW may be specific to daily oral PrEP as a delivery mechanism, given that taking a daily tablet is harder to hide, and that the packaging leads oral PrEP to be mistaken for ARVs. It may be that other PrEP modalities, such as injectables and vaginal rings, would circumvent challenges related to oral PrEP packaging and concerns around discretion, and thus reduce the importance of disclosure for successful persistence [37–39].

Concerns around PrEP stigma were salient in the narratives of AGYW in all groups. Among those currently taking PrEP, concerns around PrEP stigma related to the packaging of oral PrEP tablets, difficulties in disclosing PrEP use, or negative reactions after disclosing. These barriers however were not sufficient for these AGYW to discontinue. Among those who discontinued, PrEP stigma was a major issue and related to the physical packaging of oral PrEP, leading to PrEP being confused with ARVs by others, the associations of PrEP with promiscuity, and being accused by partners who saw PrEP use as a sign of infidelity. Among refusers, concerns about anticipated PrEP stigma related mostly to the worry that PrEP would be confused with ARVs by others. Clearly, without issues related to PrEP stigma being addressed, PrEP use and persistence will be impeded. Suggestions to reduce PrEP stigma include the need to shift the narrative surrounding PrEP away from being associated with the risk discourse, which is tied to perceptions of promiscuity and deviance, towards one of responsibility and empowerment [24].

Framing and social marketing of PrEP as one component of an integrated package of SRH self-care products that empowered AGYW can use, alongside contraceptives and condoms, could help to normalise PrEP, promote discretion and self-efficacy, mitigate issues related to anticipated PrEP stigma and thus boost uptake and persistence [8, 24, 26, 36]. Additionally, tying in the provision of contraceptive and PrEP modalities with similar patterns of return visits provides a further opportunity to integrate SRH services, normalise PrEP and enhance accessibility [39]. As long-acting PrEP options become increasingly available, lessons can be learned from contraceptive service provision in which health care providers offer informed choice counselling and use decision-making tools to support individuals to compare available options and consider their unique needs and preferences at a given time point [38, 39]. Similarly, PrEP decision support tools should provide information about the different PrEP modalities.

### Limitations

The findings pertain to AGYW’s views and experiences of any PrEP services in their community, and not necessarily those connected with the My Journey Programme. In many cases respondents were not able to recall the name of the programme or organisation offering PrEP services. However, all interview respondents were recruited from communities in which the intervention had been implemented, and had indicated during the survey that they had participated. Our sample was limited in that it did not include AGYW who had started, stopped, and started on PrEP again. In future studies it would be useful to purposively sample AGYW who had re-initiated PrEP use. Although the sample included adolescent girls in the 15–17 years age group, most of them had refused PrEP so were not represented in currently using and discontinuer categories. Additionally, due to challenges recruiting girls in this age group, there were relatively few adolescent girls interviewed as compared to young women in the older age group.

### Conclusions and Implications

Decision-making and behaviours relating to HIV prevention are situated within an ecosystem and influenced by multi-level factors and interactions [11, 26, 36]. HIV prevention interventions should incorporate choice counselling to enhance and support informed PrEP decision-making, taking into consideration individuals’ preferences, values, goals, motivations and life circumstances [25, 40]. It is clear that in order to address barriers to consistent PrEP use and persistence amongst South African AGYW, innovative and tailored interventions are needed [38]. In order to enhance and support AGYW agency in their HIV prevention decision-making, PrEP counselling and service provision need to be client-centred, youth friendly, non-judgemental, and responsive to AGYW emotions, motivations, risk perceptions, relationships and life situations and the way in which they shift and are impacted by contextual factors [18, 38, 41, 42]. It is critical that PrEP services account for the dynamic nature of decision-making, and the fluidity of PrEP users’ relationships, risk perceptions and motivations, as well as the interpersonal contexts in which they are situated [11].

Positioning HIV prevention within this ecological framework underscores that sustained PrEP use cannot be achieved through individual-level interventions alone. Interventions must integrate strategies that promote emotional resilience and hope, strengthen family and partner support networks, normalise PrEP through positive community framing, and reform health systems to ensure confidential, responsive service delivery. Incorporating choice counselling and multimodal PrEP options will be essential for enabling informed, confident, and contextually supported decision-making among AGYW.

## Supplementary Information

Below is the link to the electronic supplementary material.

Supplementary Material 1

## Data Availability

The qualitative datasets generated and analysed during the current study are not publicly available due to the potential to violate the privacy of the human subjects, but are available from the corresponding author on reasonable request.
