# Supplementary material for: “I Just Stopped Taking PrEP Without a Reason”: Understanding PrEP Decision-Making Amongst South African Adolescent Girls and Young Women (AGYW)
Source: AIDS Behav. 2025 Dec 21;30(11):3288–99. doi: 10.1007/s10461-025-04994-3 (PMC13727867; doi:10.1007/s10461-025-04994-3)
Supplement: Supplementary file 1 [file 10461_2025_4994_MOESM1_ESM.docx]

**Supplementary Information**

***Interview Guide questions relating to PrEP***

**PrEP**

Now let’s discuss PrEP

- **(for all AGYW)** What do you know about PrEP?
  - Where did you hear about PrEP from?
    - Probe: social media, other media (TV, radio etc), peers, influencers, teachers, safe spaces, mobile clinics, parents/caregivers, community members, other
    - What did you hear about PrEP?
  - Were you ever offered PrEP in the programme?
    - What were you told about PrEP when you were offered it?
    - What were your feelings and thoughts about PrEP at the time?
      - Positive feelings
      - Negative feelings
  - Have these feelings changed over time – why / how?
- **(for HIV negative AGYW)** Are you currently using PrEP?
  - **If yes**, could you tell me about your experience using PrEP?
    - What made you decide to take PrEP
      - Was there anyone who helped you to make this decision?
    - How do you feel about using PrEP?
      - What things do you like about it?
      - Are there things you don’t like about it?
      - Do you plan to continue using PrEP?
        - What would make you want to continue using it?
        - What would make you want to stop using it?
  - Could you tell me about any discussions you’ve had about using PrEP.
    - Have you discussed this with your partner, if you have one?
      - If yes, what was their reaction?
      - If no, are there reasons you haven’t discussed it with them?
      - What makes is easy or difficult to discuss your PrEP use with a partner?
      - What would make it easier?
    - Have you discussed this with a parent or caregiver?
      - If yes, what was their reaction?
      - If no, are there reasons you haven’t discussed it with them?
      - What makes is easy or difficult to discuss your PrEP use with parent or caregiver?
      - What would make it easier?
    - Have you discussed this with peers/friends?
      - If yes, what was their reaction?
      - If no, are there reasons you haven’t discussed it with them?
      - What makes is easy or difficult to discuss your PrEP use with a friend?
      - What would make it easier?
    - Are there people you have chosen not to discuss your PrEP use with?
    - Where did you get PrEP from?
      - How was your experience getting it?
      - What was the person who gave it to you like?
      - How did you feel at the time you were getting it?
      - Are there things that made your experience good?
      - Are there things that would have made your experience better?
- **If you have been offered PrEP but are not using it**, could you share with us the reasons you have decided not to use it?
- **For those not using PrEP**, could you tell us about any experiences you’ve had discussing PrEP with other people?
  - Probe:
    - Parents / caregivers
    - Friends / Peers
    - Partners
    - Anyone else
